# IgG4-related autoimmune pancreatitis mimicking a degenerating intraductal papillary mucinous neoplasm

**DOI:** 10.1055/a-2849-5935

**Published:** 2026-04-27

**Authors:** Gemma Rossi, Marco Schiavo Lena, Maria Chiara Petrone, Giulio Belfiori, Emanuel Della Torre, Massimo Falconi, Paolo Giorgio Arcidiacono

**Affiliations:** 1 Pancreato-Biliary Endoscopy and Endosonography DivisionPancreas Translational & Clinical Research Center, San Raffaele Scientific Institute IRCCS, Vita-Salute San Raffaele UniversityMilanItaly; 2 Pathology UnitPancreas Translational & Clinical Research Center, San Raffaele Scientific Institute IRCCS, Vita-Salute San Raffaele UniversityMilanItaly; 3 Division of Pancreatic SurgeryPancreas Translational & Clinical Research Center, San Raffaele Scientific Institute IRCCS, Vita-Salute San Raffaele UniversityMilanoItaly; 4 Unit of Immunology, Rheumatology, Allergy and Rare DiseasesPancreas Translational & Clinical Research Center, San Raffaele Scientific Institute IRCCS, Vita-Salute San Raffaele UniversityMilanItaly

Autoimmune pancreatitis (AIP) represents a benign inflammatory disease, with two subtypes and specific histological and serological features (IgG4 presence in the tissue) 1 . In contrast, intraductal papillary mucinous neoplasia (IPMN) represents a potential malignant cystic disease, with a range of benign, borderline (the presence of worrisome features or high risk stigmata) or malignant disease manifestations 2 .

Sometimes these two entities can present themselves with unusual clinical manifestations and endoscopic ultrasound (EUS) represents the choice exam to high-accurate diagnosis in both diseases 3 .

A 54-year-old man with a systemic hypereosinophilia conditioning renal and pulmonary manifestations presented in our referral hospital for pancreatic diseases to perform an EUS with the suspect of a degenerated IPMN, with multiple nodules projecting into the main pancreatic duct (MPD) already evaluated and biopsied in another center resulting in inflammatory infiltration (eosinophils, plasma cells and lymphocyte infiltration, negative for IgG4 immunohistochemistry) and studied at a previous magnetic resonance.

**Fig. 1:**
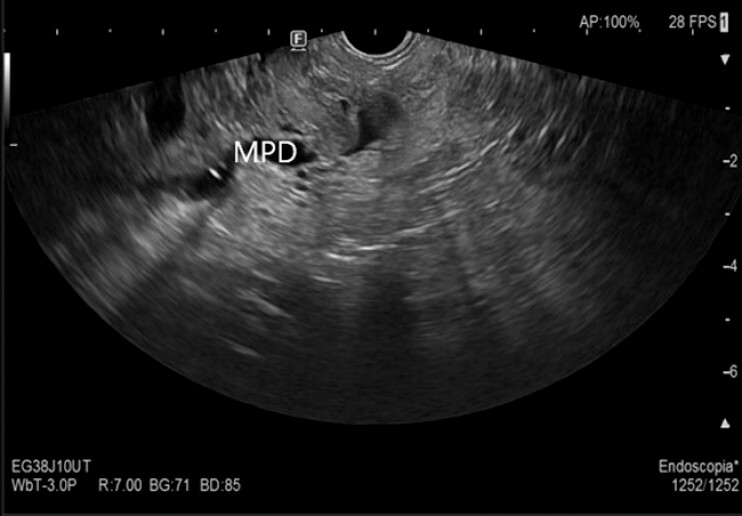
Intraductal pattern growth in the main pancreatic duct (MPD) B-mode aspect of the major nodule.

**Fig. 2:**
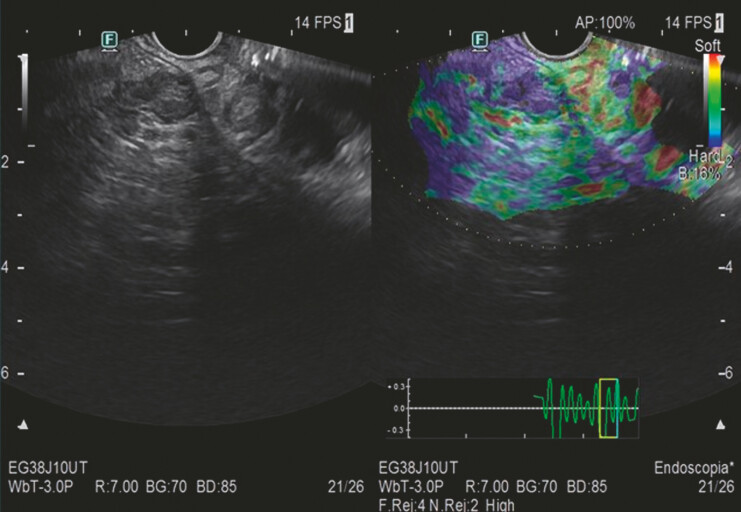
The elastography aspect of an intraductal nodule in a branch pancreatic duct.

EUS revealed a solid, hypoechoic, rigid, vascularized lesion located filling the MPD at the level of pancreatic body causing upstream ductal dilatation (up to 6 mm in the tail). The dilated MPD was also communicating with a branch-duct cyst, suggestive of a mixed-type IPMN ( Fig. 1 ). This nodule was suggestive after the intravenous contrast injection for a solid-vascularized lesion, with an early enhancement in the arterial/early phase and a slow wash-out in the venous/late phase ( Video 1 ). Other smaller cysts with solid material inside were evidenced along the pancreatic gland ( Fig. 2 ). A fine needle aspiration with on-side cytologic evaluation (ROSE) of the solid lesion filling the MPD described in the pancreatic body was performed which revealed the presence of an IPMN with low grade dysplasia.

The B-mode aspect of the pancreatic nodule with intraductal pattern growth in the main pancreatic duct (MPD) and branch ducts with vascularization of the nodule at contrast intravenous injection.Video 1

**Fig. 3:**
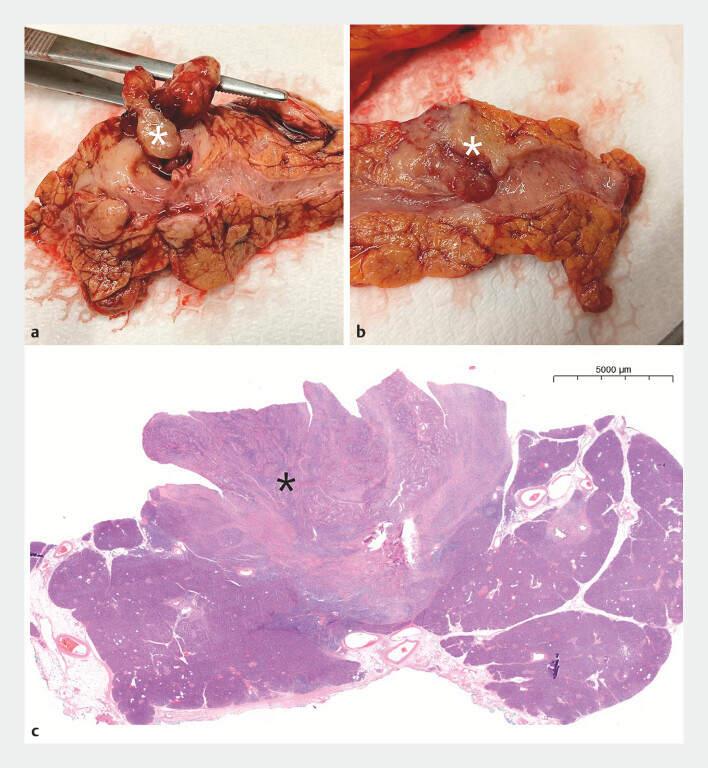
A polypoid formation protruding into the lumen of the main pancreatic duct is visible either on gross ( **a** and **b** , white asterisk) and on histological examination ( **c** , black asterisk).

**Fig. 4:**
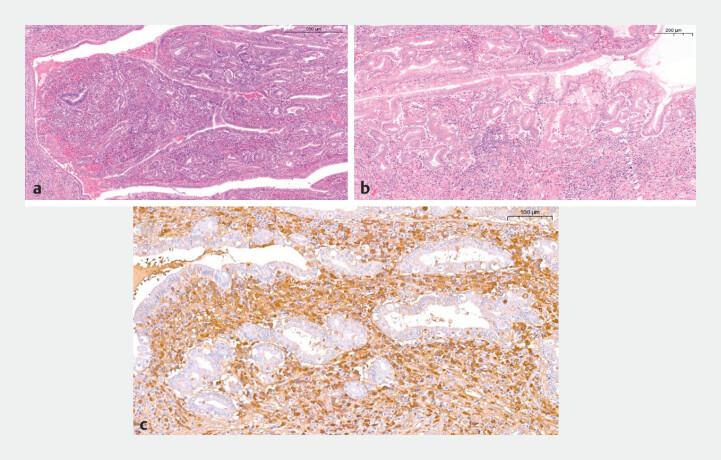
The axis of the polypoid formation consists of a dense inflammatory lymphoplasmacytic infiltrate with numerous eosinophils ( **a** and **b** ) and IgG4+ plasma cells ( **c** ). The count of the latter was >100 cells/HPF with an IgG4/IgG ratio of up to 40%.

The patient underwent surgical resection (distal splenopancreatectomy) and definitive histology described the intraductal nodule of the pancreatic body as a type-1 autoimmune IgG4 related pancreatitis with a rich eosinophilic infiltrate associated with a low-grade dysplasia branch duct IPMN. Plasma cells IgG4+ were numerous in the tissue but the serological dosage of IgG4 was negative ( Fig. 3 , Fig. 4 ). The patient is now undergoing Rituximab infusions (two infusions performed) and no imaging has been performed yet.

AIP could present atypical manifestations and can also mimic potentially malignant disease like IPMN. Although EUS represents the method of choice for pancreatic masses diagnosis, in this specific case the final histological evaluation of the surgical sample resulted in a unique and final way to reach the correct diagnosis. Finally, the study of the case in a referral center with an expert team in pancreatic diseases is fundamental to establish the correct management, diagnosis and therapy.

Endoscopy_UCTN_Code_CCL_1AF_2AZ_3AB
